# Anatomical changes in the male pelvis between the supine and upright positions—A feasibility study for prostate treatments in the upright position

**DOI:** 10.1002/acm2.14099

**Published:** 2023-07-24

**Authors:** Andries (Niek) Schreuder, Wen‐Chien Hsi, John Greenhalgh, Michael Kissick, Michelle Lis, Tracy S. A. Underwood, Harry Freeman, Michael Bauer, Stephen Towe, Rockwell Mackie

**Affiliations:** ^1^ Leo Cancer Care Middleton Wisconsin USA; ^2^ University of Arkansas for Medical Sciences (UAMS) Department of Radiation Oncology Little Rock, Arkansas USA; ^3^ Fonar Corporation Melville New York USA; ^4^ Leo Cancer Care Horley Surrey USA

**Keywords:** anatomical changes, upright imaging, upright radiation therapy

## Abstract

Treating and imaging patients in the upright orientation is gaining acceptance in radiation oncology and radiology and has distinct advantages over the recumbent position. An IRB approved study to investigate the positions and orientations of the male pelvic organs between the supine and upright positions was conducted. The study comprised of scanning 15 male volunteers (aged 55–75 years) on a 0.6 T Fonar MRI scanner in the supine and upright positions with a full bladder and in the upright position with an empty bladder.

The Pelvic study revealed that in the upright position the

1. Position and shape of the prostate are not impacted significantly by bladder fill.

2. Distance between the sacrum and the anterior bladder wall is significantly smaller.

3. Anterior‐Posterior length and the bladder width is significantly larger.

4. Seminal vesicles are pushed down by the bladder.

5. Top of the penile bulb is further away from the apex of the prostate.

These observed differences could positively impact upright prostate treatments by

1. Reducing the risk of small bowel approximating the treatment volume.

2. Prostate treatments can be done with a reduced focus on bladder fill.

3. Radiation beams for treating intermediate risk prostrate can be made smaller or a larger portion of the seminal vesicles can be treated with the same beam size than typically used for supine treatments.

4. Reducing the average dose to the penile bulb.

## 1 INTRODUCTION

Treating patients in the upright position using external beam radiation therapy has a long history. Two recent papers from Sulman et al.^1^ and Volz et al.^2^ provide summaries of the initial uses of upright treatments in the early days of radiation therapy. When CT scanners were introduced to the market circa 1975, external beam radiation therapy moved to a model of treating patients in a supine posture to match the position in which the CT images were acquired.^1^, ^2^ Several attempts were made in the 80′s to develop upright CT scanners to scan patients in either a standing position for treatments using the Fermilab fixed horizontal neutron beam^3^, ^4^ or in the seated position for particle therapy at the Lawrence Berkeley Laboratory (LBL),^5^, ^6^ the Harvard Cyclotron Laboratory (HCL)^7^ and the HIMAC facility in Japan.^8^ One major problem with the earlier systems was that the scan times were very long, mainly due to the infancy of the CT and computer technologies and the fact that continuous helical scanning was not yet available. The very long scan times resulted in unwanted patient motion during the CT scanning. It was surmised that patients could tolerate longer scan times better in a supine position. Patient fatigue was blamed for the virtual abandonment of upright imaging for radiation therapy planning by 2003.

Nevertheless, the seated position is still used regularly for intracranial treatments at many particle therapy facilities.^9^ ^,10^ Several recent papers address the benefits and possible advantages of upright treatments and the rationale to re‐consider treating patients in the upright orientation again.^1^, ^2^, ^11^, ^12^, ^13^ Treating abdominal and pelvic organs in the upright position requires that the imaging be done in the same orientation since the impact of the gravitational changes on the organs will displace and change the shape of organs such as the bladder, liver, spleen, pancreas, the large and small bowel.^14^, ^15^, ^16^, ^17^ A search of the published literature revealed a lack of data regarding organ changes between supine and upright positions in the male pelvis. For females, pelvic organ prolapse (POP) is a common medical problem and has been researched extensively using upright MRI scanners.^18^

A recent paper by Boisbouvier et al. showed excellent results for immobilizing the pelvis in the upright orientation using external surface data.^19^ State of the art CT scanners are also much faster. Jinzaki et al. reported on a significant reduction in overall scan times in the upright orientation.^16^ However, the internal pelvic anatomy is of extreme importance in treating pelvic organs such as the prostate gland. The prostate gland is situated between the bladder, the rectum, and the pubic symphysis joint. The primary challenge in prostate treatments is to minimize the radiation dose to the femurs, rectum, penile bulb, seminal vesicles, and bladder while considering displacements of the prostate due to changes in rectal content and bladder fill. Another problem that often arises is the proximity of the small bowel to the prostate which, in some patients, is mostly related to anatomical anomalies. The prostate can move as much as 10 mm in both the superior‐inferior (SI) and anterior‐posterior (AP) directions.^20^ It is expected that random bowel movements cause most prostate displacements. It is unclear how that dynamic will change for the upright patient orientation and needs to be investigated.

Techniques have been developed to reduce the radiation dose to neighboring tissues when treating the prostate in a recumbent position. The use of SpaceOAR Gel^21^, ^22^ was introduced in 2016 to create space between the rectum and prostate in order to reduce the dose to the anterior rectal wall. Additionally, treating the patient with a semi‐full bladder stretches the bladder wall, moving most of the bladder wall away from the radiation beams. Using a rectal balloon does the same for the rectal wall. A full bladder also creates more distance between the sigmoid colon, bowel and the prostate by pushing these structures more inferior to the prostate. However, being treated with a full bladder is not physically or emotionally comfortable for the prostate patient undergoing treatment. A patient who is anxious about urinary incontinence may fidget, resulting in setup error. Fujioka et al. reported on the optimal bladder volume for patients undergoing prostate volumetric arc therapy stressing the importance of reproducing the bladder volume at the time of acquiring the treatment planning CT scan.^23^

Changes in rectal content, that is, mainly rectal gas but also stool, can rapidly change the displacement of the prostate in the recumbent position. The appearance (formation) of gas in the rectum has been addressed using a special rectal balloon that has an air release mechanism that allows for rectal air to escape after the balloon has been inserted. The use of a full bladder and a rectal balloon also anchor the prostate in a more stable position during treatment by applying pressure on the prostate in two directions.

Considering the forementioned challenges, the question therefore arises whether it is even feasible to treat the prostate in the upright position based on the possible anatomical changes in the male pelvis between the recumbent and upright positions. The goal of the present work is to answer this question by looking at the changes in the prostate position, bladder shape and changes in anatomical distances between the major pelvic structures between the supine and upright orientations. It is postulated that the increased gravitational forces on the pelvic organs in the upright orientation will stabilize and anchor the pelvic organs, more specifically the prostate, and will have a net positive impact towards prostate treatments in the upright position.

## 2 MATERIALS AND METHODS

### 2.1 Anatomical study

A study to observe and measure the anatomical changes in the male pelvis between the supine and upright positions using an upright MRI scanner was conducted. The study was designed based on a preliminary study that investigated changes in the prostate position between the supine and upright positions, using an upright MRI scanner, as was reported by Mackie et al.^11^ IRB approval was obtained to study 15 volunteers between 55 and 75 years of age *(Protocol LCC‐001, WCG IRB Protocol ID 20211980)*. Fifteen volunteers with intact prostates were recruited into the study and men that had previous prostate procedures such as radiation therapy to the pelvic region (external beam or brachy therapy) or ablative procedures were excluded. All participants underwent MRI scans on a FONAR 0.6 T UPRIGHT® MRI at FONAR headquarters in Melville, New York. The UPRIGHT® MRI is equipped with an adjustable patient bed that enables scanning patients at bed angles ranging from 0 degrees (horizontal) to 84 degrees (near vertical). Each volunteer spent about 45 min at the facility. The MRI study included T2‐weighted sagittal and axial scans with a semi‐full (at least 70%) bladder in the supine position. Each volunteer was then repositioned in the same scanner, and the scans were repeated in the upright position. The volunteer then went to the bathroom and emptied his bladder. After voiding, the volunteer returned to the scanner for the same T2‐weighted axial and sagittal scans with an empty bladder in the upright position. Sixteen sagittal T2‐weighted images were acquired in 1:35 using a driven‐equilibrium fast spin echo (FSE) sequence (TR/TE = 3968.0 ms/120 ms, NSA = 1) with 2.0 mm × 2.0 mm in‐plane resolution and a 5.0‐mm slice thickness (inter‐slice gap of 1.0 mm). Forty axial T2‐weighted images were acquired in two interleaved 4:56 acquisitions using a FSE sequence without driven equilibrium (TR/TE = 4630.1 ms/120 ms, NSA = 3) with 2.0 mm × 2.0 mm in‐plane resolution and a 3.0‐mm slice thickness (no gap between slices). The displayed field‐of‐view was 260 mm in both cases. The objectives of the scan protocols are listed in Table 1.

**TABLE 1:** Scan type, patient position and bladder fullness, and scan objective.

MRI scan type	Patient position—bladder fill	Objective
T2‐weighted axial and sagittal scans	Supine—Full Bladder	Establish detailed pelvic organ shape and position in supine position.
T2‐weighted axial and sagittal scans	Upright—Full Bladder	Establish detailed pelvic organ shape and position in upright position.
T2‐weighted axial and sagittal scans	Upright—Empty Bladder	Evaluate the impact of bladder fill on the prostate shape and position.

**FIGURE 1:**
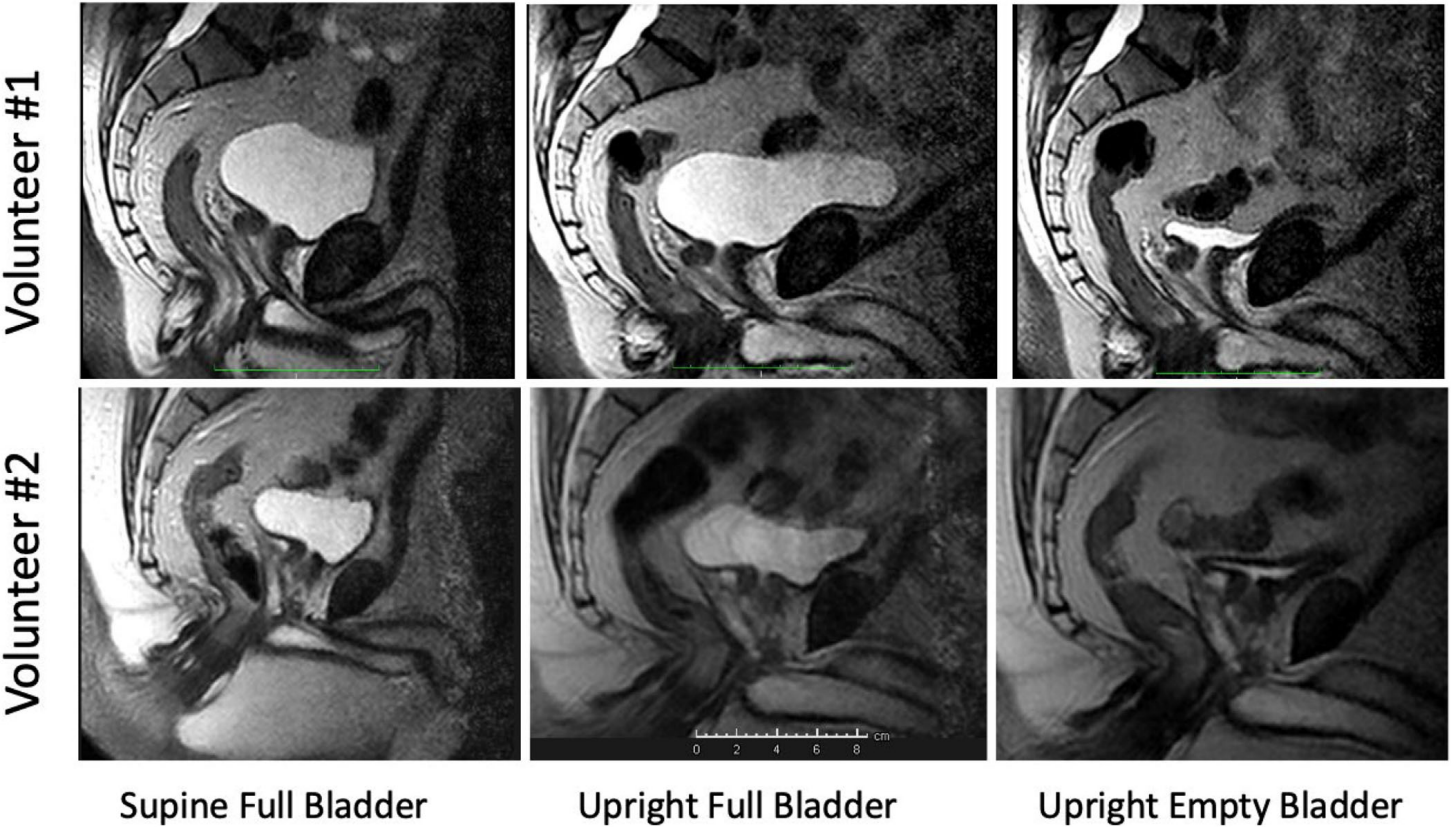
T2‐weighted sagittal MRI images in the pelvic region of Volunteer 1 (top row) and Volunteer 2 (bottom row) in each of the three conditions.

Figure 1 displays representative midplane sagittal images for two volunteers in each of the three conditions listed in Table 1.

**FIGURE 2:**
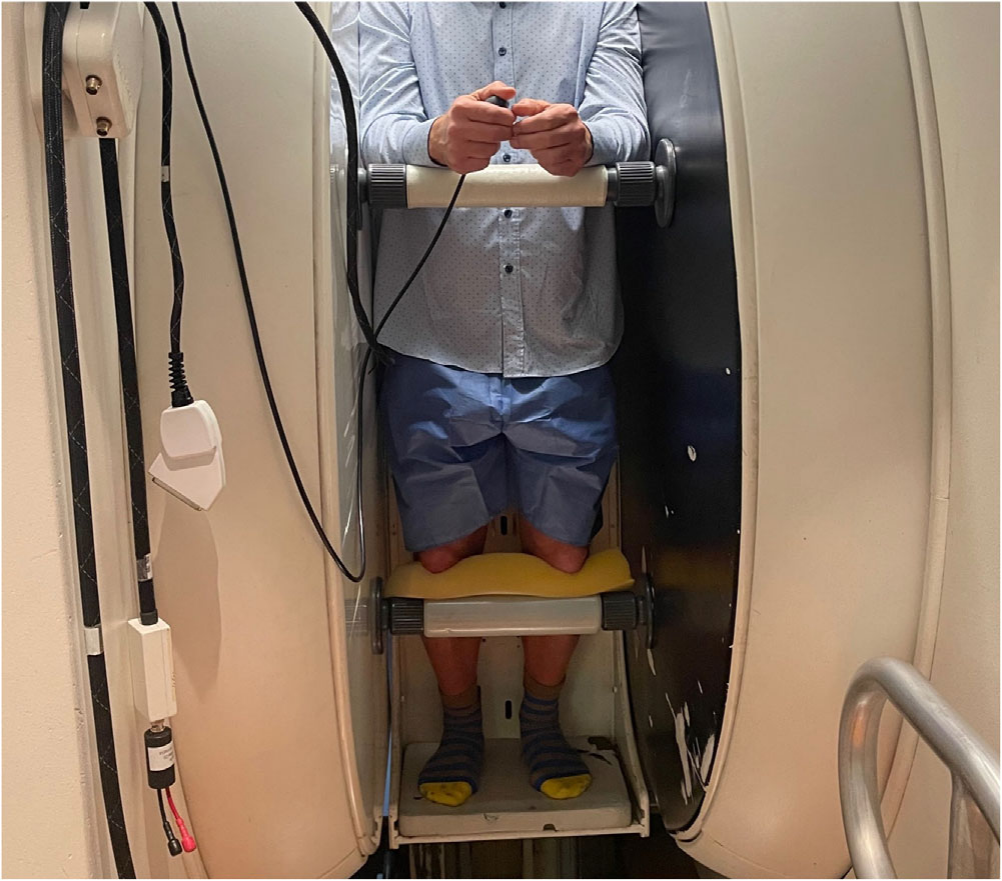
A volunteer in the MRI scanner demonstrating the upright position. Trans‐polar VersaRests™ were positioned to support the knees and arms.

T2‐weighted sequences were chosen for this study because they produce bright signals from urine in the bladder and excellent contrast between surrounding soft tissues and landmark bony structures, as seen in Figure 1. The scan parameters (in‐plane resolution and slice thickness) were chosen to afford good geometrical definition of soft tissues in the prostate region in reasonably short scan times. For patient comfort, a soft pillow was placed under the knees of each volunteer in the supine position. For the upright scans the patient support table was rotated to an angle of 84 degrees, that is, 6 degrees off vertical, and two support bars (VersaRests™) were inserted between the magnet pole covers, one as a knee stop and one as an arm rest. Neglecting to also insert a support bar under the buttocks was a mistake since resting only the knees on the support bar became uncomfortable for some volunteers and resulted in unwanted motion during the scan. A picture of one of the volunteers in the upright position is shown in Figure 2. The knee and arm supports can be seen in the photograph.

### 2.2 Data analyses

#### 2.2.1 Anatomical landmarks

**FIGURE 3:**
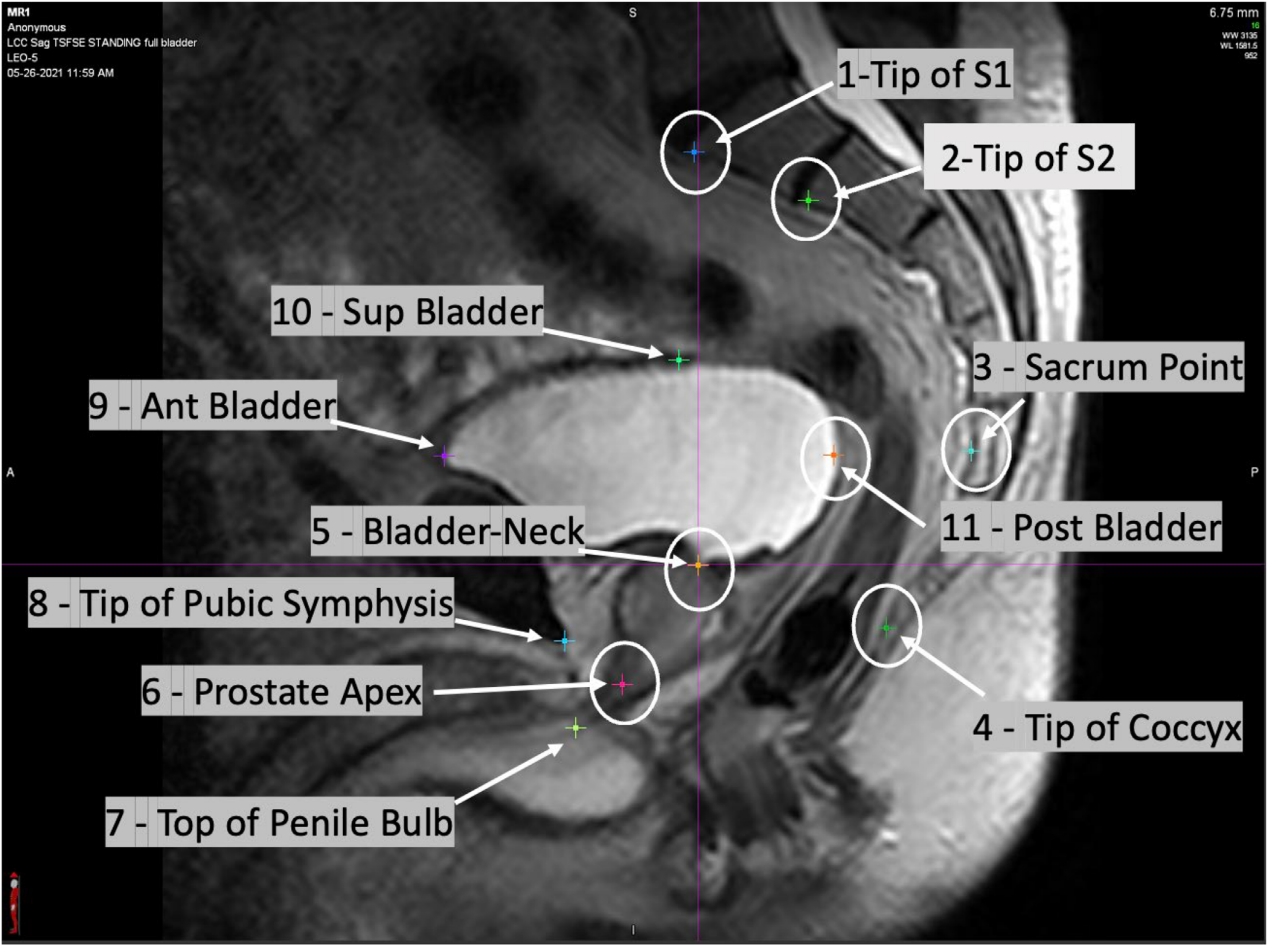
The anatomical landmarks identified in the central sagittal plane for one of the volunteers in the upright position with a semi‐full bladder.

The scans for all the volunteers were analyzed in several ways using the MIM Maestro V6.9.3 platform (www.mim‐software.com). Discrete anatomical landmarks for all the volunteers using the sagittal and axial scans were identified. The 3D coordinates of all the landmarks were exported from MIM using a DICOM format and were used to calculate several metrics to evaluate the anatomical changes between the supine and upright orientations. The anatomical landmarks identified in the central sagittal plane are illustrated in Figure 3 for one of the volunteers in the upright position with a semi‐full bladder.

#### 2.2.2 Corrections for pelvic rotation

**FIGURE 4:**
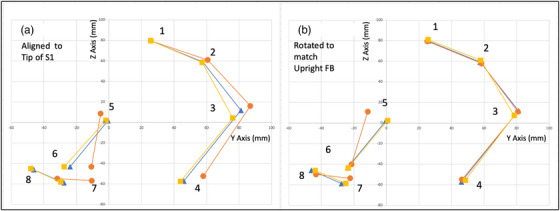
The coordinates for some of the anatomical landmarks in the sagittal plane of one of the volunteers. Three sets of points are plotted for each volunteer, corresponding to the three conditions: circles: supine full bladder; triangles: upright full bladder; squares: upright empty bladder. The left panel shows the coordinates aligned to the “Tip of S1” point for all three conditions. The right panel shows the coordinates after rotation and translation of the supine full bladder and upright empty bladder points based on aligning the spinal axis points to the upright full bladder points using a least‐squares minimization technique. The specific landmarks are numbered according to the numbers and names illustrated in Figure 3.

Anatomical landmarks along the spinal axis, that is, Tip of S1, Tip of S2, Sacrum Point, and the Tip of Coccyx were used to calculate and correct for pelvic rotations between the different scans for each volunteer. The alignment was based on a least‐squares optimization, minimizing the distances between the corresponding landmarks using rotations and translations in the sagittal plane only. The Y and Z DICOM coordinates for some of the anatomical landmarks for a randomly selected volunteer are plotted in Figure 4. The positive Y axis is posterior to anterior, and the positive Z axis is superior to inferior. Panel A in Figure 4 shows the data points aligned to the “Tip of S1” for all the scan conditions. The data points, aligned to the upright full bladder condition for the same volunteer, are shown in panel B of Figure 4. The transformation parameters obtained during the alignment of the spinal axes were then applied to the other anatomical landmarks for the supine full bladder and upright empty bladder conditions. The transformed landmarks relative to the upright full bladder orientation were then used to calculate the required metrics, listed in Table 2, to determine whether significant changes occurred between the supine and upright orientations.

**TABLE 2:** The respective metrics calculated using certain anatomical landmarks.

Metric	Anatomical points used
Posterior bladder to sacrum distance	3 + 11
Tip of S1 to bladder neck distance	1 + 5
Distance between the bladder neck positions	5
Distance between the prostate apex positions	6
Anterior to posterior (AP) length of the bladder	9 + 11
Prostate apex to top of penile bulb	6 + 7
Pelvic rotations	From transformation matrix
Prostate rotations	Angle between Z axis and the vector connecting points 5 + 6

#### 2.2.3 Calculation metrics and methodologies

The different metrics listed in Table 2 were calculated using the transformed anatomical landmarks to investigate whether significant changes between the specific landmarks for the supine and upright orientations exist. More details about each of the metrics are described in Appendix A.

#### 2.2.4 Changes in the prostate position

The change in position of the prostate between the supine and upright positions was characterized by calculating the vector lengths between the bladder neck positions in the supine and upright orientations with a full bladder and the upright orientation with a full and empty bladder respectively. The same calculations were done for the prostate apex positions.

#### 2.2.5 Rotation of the prostate

With the scans aligned to the full bladder upright scans it was possible to examine the rotation of the prostate in the sagittal plane between the scans. The bladder neck and apex positions were used to calculate the rotation angle of the prostate. The rotation angle was calculated using Equation (1) relative to the full bladder upright orientation which was used as the reference position.

(1) $$\begin{array}{c} \mathrm{Rotation}\;\mathrm{Angle}=\mathrm{ARC}\;\mathrm{TAN}\left(\frac{\left(Y_{\mathrm{Ref}\left(\mathrm{Bneck}\right)}-Y_{\mathrm{Apex}}\right)}{\left(Z_{\mathrm{Ref}\left(\mathrm{Bneck}\right)}-Z_{\mathrm{Apex}}\right)}\right) \end{array}$$

The Y and Z coordinates for the supine full bladder and the upright empty bladder were used in Equation (1) for the Y*_Apex_* and Z*_Apex_* parameters to calculate the rotation of the prostate relative to the full bladder upright position. A positive angle means an anti‐clockwise rotation relative to the upright full bladder position (viewed from the volunteer's right side).

#### 2.2.6 Statistical analyses

A few types of statistical tests were performed to assess the significance of observed changes as described by Rosner.^24^ All measurements, differences, and vector changes were checked with a linear correlation between the sample quartiles and the corresponding normal curve quartiles (Q‐Q probability plot correlation coefficient), and all cases had that correlation >90%. Even though this method of checking normality amounts to a quantification of the Q‐Q plot visual inspection, it is still considered a valid check.^25^ With the data best described by paired two‐sample data, both a 2‐sided standard paired t‐test (PTT) and a Wilcoxon Signed Rank Sum Test were used with a Gaussian Approximation (WRGA) for these paired data. The WRGA, is a calculation that handles deviations from data distributed as a Gaussian better than the standard PTT. These tests arrive at a p‐value in very different ways and serve to validate one another. Technically, at least 16 data entries would be needed for the rank sum to be Gaussian distributed in all cases. The p‐values are presented according to standards.^26^ The PTT uses a Gaussian data set directly, and the WRGA uses the Gaussian distribution of the sampling ranks, The same ranks as in the standard Wilcoxon test. Therefore, the WRGA test will be valid with *N* = 15. When the data is best described by a single sample, then a one sample t‐test (OTT) was performed, 2‐sided. The vector position changes that are all positive vector modulus values were checked for significance above random chance with a Chi‐squared test (CHS). The Chi‐squared test provides a right‐sided probability that the observed variations are just by chance alone as one would expect for a normal distribution, or whether something else other than chance is at play if the test fails and the variance exceeds an expectation value that is based on chance from normal variations alone.  Hence, a small *p*‐value here confirms the expectation of the normal variation expectation of change. All the other tests had a hypothesized change of zero as the null hypothesis. Therefore, if a significance test passes (0.05 was used for the significance parameter for all tests), then it means a change that is not expected from just measurement variations are observed. If a significance test fails, it should be interpreted as not seeing evidence in these data for a significant change from the change in conditions. Two conditions were studied for significance: (1) bladder fill state: full versus empty, and (2) the patient position, upright versus supine.

## 3 RESULTS

The MRI scans for most of the volunteers showed the same interesting trend, that is, that the AP lengths of the bladder are significantly longer in the upright position (*p* < 0.001, PTT and *p* < 0.001, WRGA) and that the distances between the posterior edge of the bladder and the sacrum are significantly smaller in the upright position (*p* = 0.0032.6 × 10^−3^, PTT and *p* < 0.001 = 8 × 10^−4^, WRGA).

**FIGURE 5:**
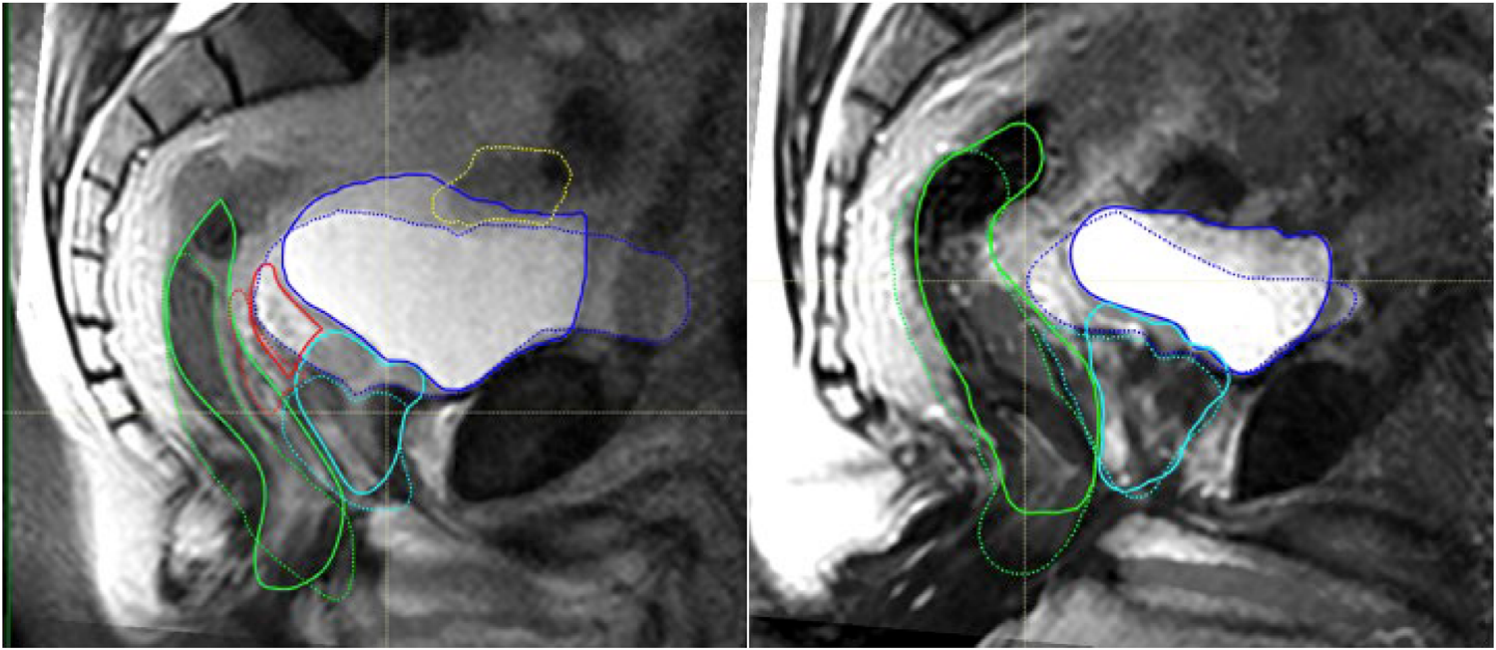
Midline sagittal MRI images for two volunteers in the supine and upright full bladder conditions. Superimposed on the images are contour lines delineating the organs in the supine (solid lines) and upright (dashed lines) positions. The following organs are also shown in both panels—rectum (green), prostate (light blue), bladder (blue) and small bowel (yellow—left panel only).

The distances between the Tip of the S1 vertebra and the bladder neck (representing the top of the prostate) for the upright full bladder orientations are significantly larger than in the supine position (*p* = 0.014, PTT and *p* = 0.011, WRGA). This SI shift of the prostate is clearly visible in Figure 5 which shows supine and upright sagittal MRI images superimposed on each other for two volunteers. The same distances for the upright full versus upright empty bladder are not significant (*p*>>0.05, OTT and *p*>>0.05 WRGA). The average change in the Tip of S1 and the bladder neck position in the upright position with a full and empty bladder is only −0.5 ± 2.1 mm.

The changes in the bladder neck and prostate apex positions with a full and empty bladder in the upright position were tested for significance. Each direction, Y and Z for the prostate apex showed no significant changes due to bladder fill in the upright position (*p*>>0.05), and the vector change also was not significant (*p*>>0.05). A Chi‐squared test with the expected variation equal to observed variations in each direction shows that changes in bladder conditions, are likely just expected variations (*p* = 0.001, CS, right tail). This is the same for the bladder neck position changes. The state of the subject's bladder filling did not have any significant effect on the bladder neck positions, in neither the Y nor the Z directions (*p*>>0.05). The vector changes in the bladder neck were also not significantly changed with respect to bladder fill (*p*>>0.05). The same Chi‐squared test as described above showed that the observed changes in bladder conditions with the bladder neck measurement are also likely just due to random variations in both directions, but since it is greater than 0.05, one could say that there are not enough measurements to conclude this with certainty (*p* = 0.092, CS, right tail).

The rotations required to align the pelvis for each volunteer for the supine full and upright empty bladder conditions to the upright full bladder orientation revealed that a significant pelvic rotation was required from supine to upright (*p* = 0.002, OTT and *p* = 0.001, WRGA).

A significant rotation of the prostate occurs in most cases between the supine and upright positions (*p* < 0.001, OTT and *p* = 0.002, WRGA). This correlates with the significant change in position of the prostate between the supine and upright orientations. The change in prostate angle as a result of emptying the bladder in the upright position was, however, not statistically significant which again correlates with the prostate position data (*p*>>0.05, OTT and *p*>>0.05, WRGA).

The distance from the apex to the penile bulb for a full bladder increased significantly when changing from the supine position to the upright orientation (*p* = 0.02, OTT and *p* = 0.02 WRGA). This average change observed in the apex to penile bulb distance was 2.4 mm ± 3.7 mm (1 SD). The same distance did not change significantly per patient between empty and full bladder conditions in the upright orientation (*p* = 0.19, OTT, *p* = 0.76 WRGA).

**FIGURE 6:**
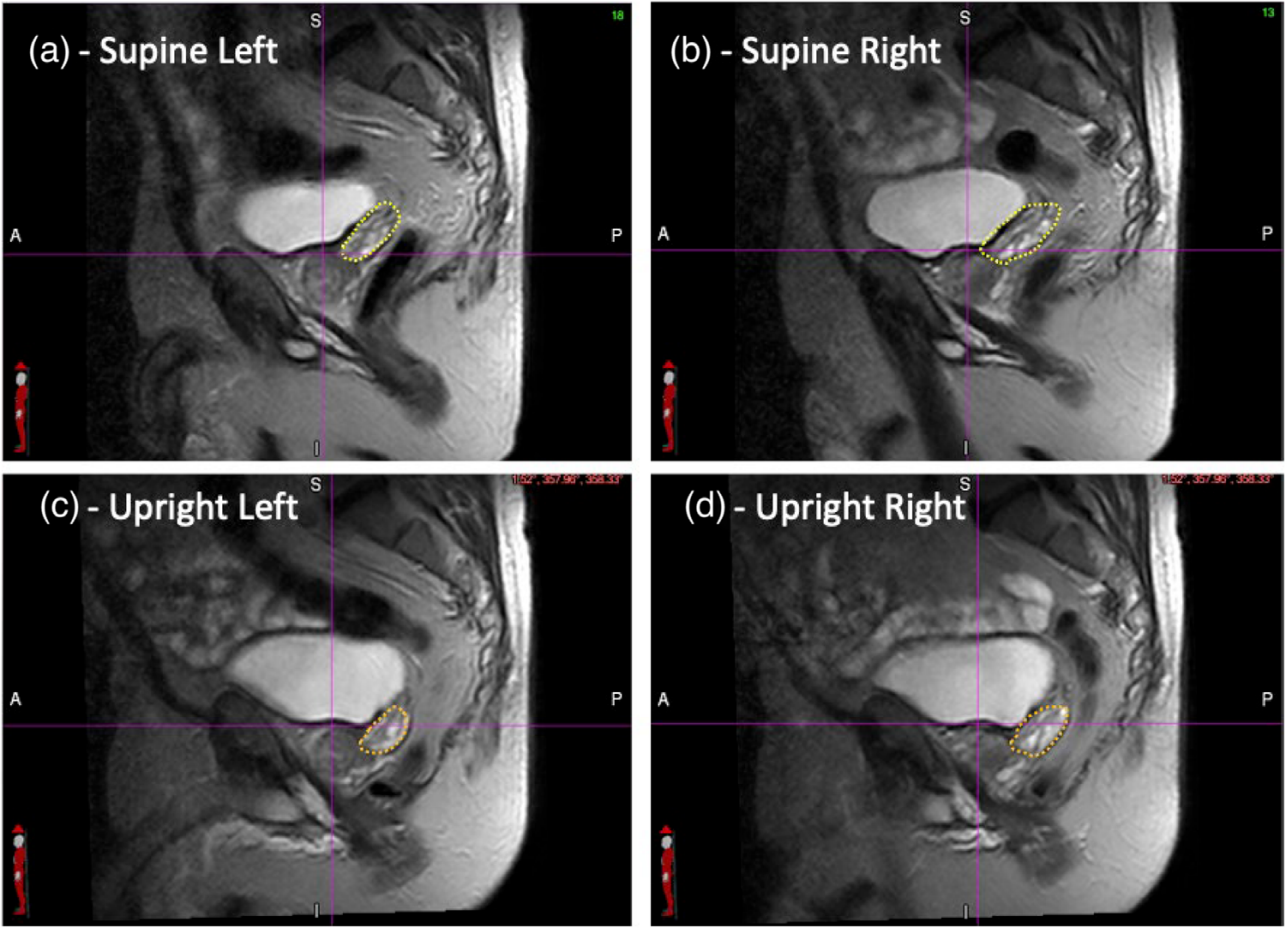
Supine and upright sagittal scans for a randomly selected volunteer showing the seminal vesicles—the yellow (supine) and orange (upright) dotted lines. The scans are 28 mm to the left and 28 mm to the right of the sagittal mid plane. The cross hairs are aligned to the bladder neck position in the sagittal mid plane.

The position and shapes of the seminal vesicles between the full bladder supine and full bladder upright orientations were also examined. A common trend was observed for all the volunteers. Since the bladder is more posterior in the upright position, the seminal vesicles appear to be pushed downwards by the larger bladder volume. This is illustrated in a Figure 6 which shows the seminal vesicles in two sagittal planes 28 mm to the left and 28 mm to the right of the mid plane for one randomly selected volunteer. The supine scans are shown in the two top panels and the upright scans in the two bottom panels. The seminal vesicles are delineated—just to guide the eye—with the yellow (supine ‐upper panels) and orange (upright—lower panels) dotted lines. The purple cross hairs in the figures are aligned to the bladder neck position on the sagittal mid plane scan.

## 4 DISCUSSION

The finding that the distance between the tip of the S1 vertebra and the top of the prostate (bladder neck) is significantly larger in the upright orientation than in the supine position, indicates that the gravitational forces on the prostate, in the upright position, pushes the prostate inferiorly—see Figure 5. The finding that the same distances are not significantly different for the full versus empty bladder conditions in the upright orientation, indicates that the SI position of the prostate, is not impacted significantly by the bladder fill condition in the upright orientation.

The finding that the shifts in both the bladder neck and apex positions are larger between the supine and upright positions than the shifts introduced because of emptying the bladder in the upright position is easy to understand bearing in mind the gravitational forces exerted on the prostate by the bladder and abdominal contents. The bladder neck points tend to shift more than the apex points which is easy to understand since the bladder is such a large organ and moves significantly in the AP direction. The apex of the prostate is further away from the bladder and is therefore less impacted by the bladder movement. This is confirmed by the rotation of the prostate relative to the prostate angle in the upright full bladder orientation calculated from the bladder neck and apex points. The large rotations in the prostate angle between the supine and upright orientations is mainly due to change in the force vector exerted on the prostate as explained above since the data was re‐aligned to correct for the natural rotation in the pelvis between the supine and upright positions. The change in the prostate angle is primarily due to the shift in the bladder neck point as discussed above.

The statistical analyses of the bladder neck and prostate apex positions in the upright orientation with a full and empty bladder, showed that there was no evidence from this study that the prostate position is affected by the bladder fill condition in the upright orientation. This is unlike the supine orientation, where the impact of changes in bladder fill, on the prostate position, is well‐known and documented.^23^, ^27^, ^28^ The insignificant impact of the bladder fill condition on the prostate position for one volunteer is illustrated in Figure 4 panel B which shows that the bladder neck and prostate apex points (points 5 and 6) are more or less the same after the data were corrected for pelvic rotation.

**FIGURE 7:**
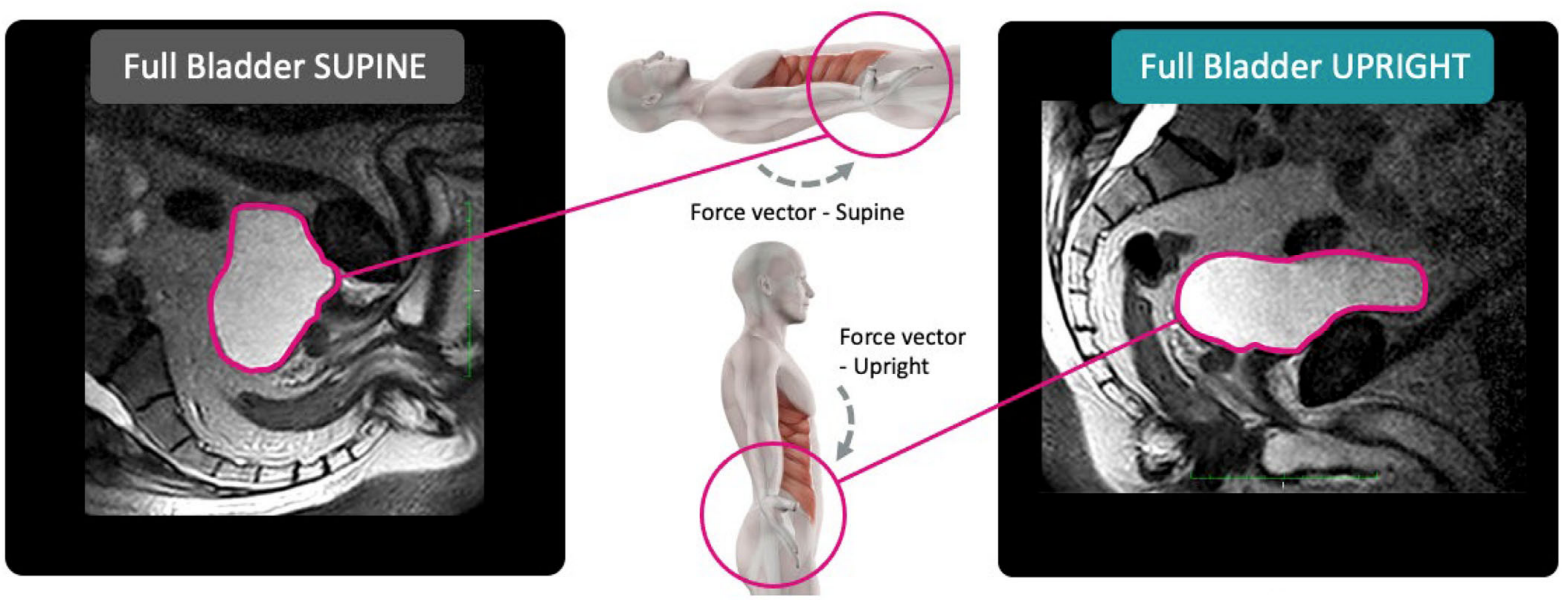
An illustration of the directions of the predominant forces (gray arrows) impacting on the bladder and prostate in the supine and upright positions. Note that the supine view on the left has been rotated 90 degrees from the usual viewing context in radiology and radiation oncology to illustrate the actual orientation of the supine patient in comparison to the upright scan.

The finding that the full bladder is significantly more elongated in the AP direction and that the posterior edge of the bladder is closer to the sacrum in the upright position can be explained by the changes in the gravitational forces on the bladder. The abdominal contents, due to gravitational forces, flop in the anterior and inferior directions (forward and downward) when the patient is in the upright position and primarily posteriorly (backwards) in the supine position. The abdominal contents therefore apply pressure onto the bladder from a more superior and anterior direction pushing the bladder down and backwards while in the supine position the force on the bladder is weaker and in a lateral direction. This is illustrated in Figure 7 showing the directions of the forces (gray arrows) impacting on the bladder and prostate in the supine and upright positions.

The fact that the posterior bladder edge is closer to the sacrum in the upright position is counter intuitive as one would think that gravity would pull the bladder more posteriorly in the supine position. This is further illustrated in Figure 5 which shows the midline sagittal supine and upright images overlaid on each other for two volunteers.

A significant pelvic rotation is evident between the supine and the upright orientations as demonstrated by the deviations between points from the supine posture to the upright posture shown in Figure 4. This can be explained looking at the changes in the total body posture between supine and upright.

The small statistically significant increase in the distance between prostate apex and the top of the penile bulb when changing from the supine position to upright orientation was a surprise. The fact that this distance was not impacted by bladder fill condition in the upright position is to be expected since the prostate position is not impacted by bladder fill. The small increase in the apex to penile bulb distance in the upright orientation could be ascribed to increased gravitational forces on the muscles inferior to the apex in the upright orientation.

The differences in the position of the seminal vesicles shown in Figure 6, might have a positive impact on intermediate risk prostate treatments in the upright position. A common practice in treating intermediate risk prostate cases is to treat only a partial volume of the seminal vesicles to reduce the dose to the rectum, bladder, and sigmoid colon. If the same approach is followed for the case shown in Figure 6, the upright orientation will allow for a much larger volume of the seminal vesicles to be treated using the same field size or that a smaller field can be used to treat the same volume of seminal vesicles. The latter means that the radiation volume could be reduced significantly thereby reducing the dose further to the critical structures.

The fact that the bladder is more elongated in the AP direction for the upright orientation, suggests that the bladder will be more effective in pushing the small bowel away from the radiation field. Furthermore, since the channel between the sacrum and the bladder is closed or narrower (see Figure 5) the chance for the small bowel to drop down into that space might be smaller.

The impact of changes in rectal content on the prostate position was not addressed explicitly in this study. However, it fair to say that there were plenty of opportunities for the rectal content to change during scans and while the volunteers left the scanner and walked to the bathroom to void the bladder. The volunteers were also not required to follow any specific dietary guidelines to limit rectal gas. Since no significant changes in the prostate position were observed between the full and empty bladder scans in the upright orientation, it is reasonable to conclude that the impact of rectal changes on the prostate position, were also reduced by the increased gravitational forces on the prostate region as described earlier. Furthermore, it might also be the case that rectal gas will tend to move more superiorly (upwards) and apply pressure more superiorly and away from the prostate. The use of a rectal spacer such as the SpaceOAR Gel to create space between the rectum and prostate will most likely be the only option for upright treatments since using a rectal balloon in the upright orientation might be problematic in many regards.

## 5 SUMMARY AND CONCLUSIONS

An IRB approved study to investigate the anatomical changes in the male pelvis between the supine and upright positions was conducted. The study revealed that the anatomical changes in the male pelvis could potentially favor the upright position for prostate treatments. The increased downward pressure on the bladder and prostate in the upright position appears to have a stabilization effect on the prostate since the changes in bladder fill did not impact the prostate position and orientation in the same manner as reported in the literature for the supine position. This holds great promise for upright prostate treatments removing the burden of a reproducing the bladder volume at the time of the CT scan.^23^

Considering the forces on the bladder and subsequently on the prostate as discussed above, it is expected that concerns about prostate movements during treatment in the upright position might be reduced or even eliminated. Since no significant changes in the prostate position were observed between full and empty bladder conditions, it is expected that the increased gravitational force on the prostate will also reduce the impact of rectal content changes on the prostate position. The fact that the bladder is more extended over the prostate and is also closer to the sacrum in the upright position might reduce the risk of small bowel getting into the treatment beams. The observation that the extended bladder in the upright orientation pushes the seminal vesicles in the inferior direction, will require a smaller radiation field to treat the same amount of the seminal vesicle for intermediate risk prostates treatments in the supine position. Although small, the increased separation between the prostate apex and the penile bulb could result in a small reduction of the average dose to the penile bulb when treated in the upright orientation. It is obvious that the empty bladder condition would not be clinically acceptable for treatment due to the proximity of the small bowel when the bladder is empty as well as the amount of bladder wall that will get irradiated.

The primary conclusion therefore is that there was nothing observed in this study of the male pelvic anatomy, that would prevent prostate treatments in the upright orientation. The preliminary data suggests some advantages in treating in upright position but more research including dosimetric comparisons when CT data of the pelvic region becomes available is recommended. Furthermore, based on the data reported by Boisbouvier et al.^19^ on immobilizing patients in the upright orientation for pelvic treatments and the fact that the changes in the bladder fill status during treatments might be less critical in the upright position, treating prostate patients in the upright orientation appears to be a promising.

## AUTHOR CONTRIBUTIONS

Niek Schreuder: Conceptualization and study design, obtained IRB approval, collected the data and wrote the paper. Wen‐Chien Hsi: Data analyses using MIM, reviewed and edit the paper. John Greenhalgh: Developed the optimal MRI scan sequences and operated the scanner. Michael Kissick: Statistical analyses of the data. Michelle Lis: Review and edit the paper. Tracy S A Underwood: Review and edit the paper. Harry Freeman: Participated in the study design, data collection and scheduling of volunteers. Michael Bauer: Participated in the study design, data collection and scheduling of volunteers. Stephen Towe: Conceptualization and study design. Rockwell Mackie: Conceptualization and study design as well as critical review and editing of the paper.

## CONFLICT OF INTEREST STATEMENT

Niek Schreuder, Michael Kissick, Michelle Lis, Tracy Underwood, Harry Freeman, Michael Bauer, Stephen and Rockwell Mackie‐ No conflict of Interest—but all are full time employees of Leo Cancer Care. Wen‐Chien Hsi—No conflict of interest. John Greenhalgh: No conflict of interest—Employee of Fonar.

## APPENDIX A

### A.1

The following metrics were calculated using the transformed anatomical landmarks to investigate whether significant changes between the specific landmarks for the supine and upright orientations exist.

2.2.2. *Posterior Bladder to Sacrum Distance (point 11 to point 3)*: Since points 3 and 11 was selected as the most posterior points of the bladder and sacrum respectively, these points are not necessarily at the same level in the superior/inferior direction (Z‐axis). Furthermore, since the objective was to determine the changes in the distance between the posterior bladder wall and the sacrum, only the Y coordinates of these points were used to calculate the distance between two planes described by the most posterior bladder edge and the sacrum point. The change in this distance was tested with the OTT and WRGA tests.

2.2.3. *Tip of S1 to Bladder Neck Distance (point 1 to point 5)*: The vector length between the superior tip of the S1 vertebra and the bladder neck was calculated for the full bladder upright and full bladder supine orientations for each volunteer. The change in this length from one patient position to another was tested with PTT and WRGA.

2.2.4. *Distance Between the Bladder Neck Positions (point 5)*: The vector lengths between the bladder neck positions for the upright full bladder and the upright empty bladder as well as the upright full bladder and the supine full bladder were calculated for each volunteer. The change in this vector length for bladder fill condition was tested with the PTT and CS tests.

2.2.4. *Distance Between the Prostate Apex Positions (point 6)*. The vector lengths between the prostate apex positions for the upright full bladder and the upright empty bladder as well as the upright full bladder and the supine full bladder were calculated for each volunteer. The change in this vector length for bladder fill condition was tested with the PTT and CS tests.

2.2.5. *Anterior to Posterior (AP) Length of the Bladder (point 11 to point 9)*: Similar to the anterior bladder to sacrum distance described in 2.2.2 above, it was not always possible to place the anterior bladder point and the posterior bladder point at the same superior—inferior (Z‐axis) level. Therefore, only the Y coordinates of these points to calculate the distance between the planes described by the anterior and posterior bladder edges (point 9 to point 11) were used. The change in AP bladder length was tested with the PTT and WRGA tests.

2.2.6. *Prostate Apex to Top of Penile Bulb*: The vector length between the Prostate Apex and the top of the Penile Bulb was calculated for the full bladder upright and full bladder supine orientations for each volunteer. The change in this length from one patient position to another was tested with PTT and WRGA.

2.2.7. *Pelvic Rotations*: The pelvic rotations between the supine and upright orientations as well as the upright full and empty bladder configuration were obtained from the transformation parameters required to align the pelvis between the three geometries studied. The observed rotations are change quantities and thus one sample data sets and was tested with the OTT and WRGA tests.

2.2.8. *Prostate Rotations*: The angle through which the prostate rotates was calculated with respect to the Z‐axis (the superior/inferior axis). The angle between the Z‐axis and the vector between the prostate apex (point 6) and the bladder neck (point 5) positions was used to approximate the rotation of the prostate. These data were also tested with both the OTT and the WRGA tests.
